# Predictors of Atrial Fibrillation in Heart Failure Patients with Indications for ICD Implantation

**DOI:** 10.3390/jcm14124358

**Published:** 2025-06-18

**Authors:** Tariel Atabekov, Roman Batalov, Evgenii Archakov, Irina Silivanova, Mikhail Khlynin, Irina Kisteneva, Sergey Krivolapov, Sergey Popov

**Affiliations:** Cardiology Research Institute, Tomsk National Research Medical Center, Russian Academy of Sciences, Kievskaya Street, 111a, Tomsk 634012, Russia; romancer@cardio-tomsk.ru (R.B.); aea_cardio@mail.ru (E.A.); panfilova.ix@mail.ru (I.S.); mskhlynin@mail.ru (M.K.); kistenevaiv@rambler.ru (I.K.); cardiorhythm@mail.ru (S.K.); psv@cardio-tomsk.ru (S.P.)

**Keywords:** heart failure, implantable cardioverter–defibrillator, atrial fibrillation, predictor, risk model, echocardiography

## Abstract

**Background/Objectives**: Atrial fibrillation (AF) is a prevalent arrhythmia that significantly complicates the management of heart failure (HF) patients, particularly those who have implantable cardioverter–defibrillators (ICDs). The interplay between AF and inappropriate ICD therapy poses a critical challenge in optimizing patient outcomes, as inappropriate shocks can lead to increased morbidity, psychological distress, and a reduced quality of life. We aimed to explore the various clinical and demographic predictors of AF in HF patients with indications for ICD implantation. **Methods**: This study included 122 patients who were indicated for ICD implantation and had undergone transthoracic echocardiography (TE). We evaluated the relationships between clinical and demographic factors and the occurrence of AF, which was recorded either before ICD implantation or during the follow-up period afterward. From our findings, we established predictors and a risk model for AF. **Results**: Out of 122 HF patients with ICDs, 52 (42.6%) experienced an episode of AF either prior to ICD implantation or during a follow-up period of 20.5 [6.0; 53.0] months, as recorded by the ICDs’ endogram. Patients with AF were older compared to those without AF (*p* < 0.001). Additionally, they exhibited a higher left ventricular early diastolic filling rate (LVE) (*p* = 0.006) and a greater left atrial index (LAI) (*p* = 0.002). These three factors—age, LVE and LAI—were found to be independently associated with AF in both univariable and multivariable logistic regression analyses. The final model, including age, LVE, and LAI, showed a good discrimination capability with an AUC of 0.775. At a cutoff value of >0.47, the model achieved a sensitivity of 67.3% and a specificity of 77.2% in identifying HF patients with ICDs at risk for AF. **Conclusions**: This study found that 42.6% of HF patients with ICDs experienced AF, with older age, higher LVE, and greater LAI identified as significant predictors.

## 1. Introduction

Atrial fibrillation (AF) is the most prevalent arrhythmia encountered in clinical practice, particularly among patients with heart failure (HF). The prevalence of AF in HF patients is significant, and it is estimated that up to 50–75% of people with severe HF may have this arrhythmia [1]. The presence of AF complicates the management of HF, as it is associated with an increased risk of adverse outcomes, including stroke, hospitalization, and mortality [2]. Furthermore, for patients with implantable cardioverter–defibrillators (ICDs), AF poses unique challenges related to device therapy.

ICDs are designed to prevent sudden cardiac death by delivering appropriate shocks in response to life-threatening ventricular arrhythmias. However, the presence of AF can lead to inappropriate ICD therapies, which occur when the device delivers shocks for non-life-threatening arrhythmias or misinterprets AF as a ventricular tachycardia (VT) event. Inappropriate shocks can result in significant psychological distress, increased morbidity, and a diminished quality of life for patients already facing the burdens of HF [2,3,4]. Understanding the predictors that contribute to the development of AF in this population is crucial for optimizing ICD programming and improving patient outcomes.

Several clinical and demographic factors have been identified as potential predictors of AF in HF patients. These include age; sex; comorbidities such as hypertension and diabetes mellitus; left atrial size; and renal function [5,6,7,8]. Additionally, the interplay between these predictors and the risk of inappropriate ICD therapy remains poorly understood. Identifying specific risk factors associated with both AF and inappropriate shocks could enable clinicians to tailor management strategies more effectively. Despite this knowledge, the relationship between these factors and AF in HF patients has not been thoroughly investigated. The aim of this study is to explore the various clinical and demographic predictors of AF in HF patients with indications for ICD implantation.

## 2. Materials and Methods

### 2.1. Study Population

A clinical, non-randomized study was conducted at the Cardiology Research Institute in the Russian Federation, involving 122 patients. Patients with indications for ICD implantation (secondary and primary prevention of sudden cardiac death) were included in the study. The study included patients who received an ICD between November 2021 and October 2024. Patients were excluded if they had experienced a myocardial infarction (MI) within the past three months, had hypertrophic cardiomyopathy, permanent AF, severe comorbid conditions (such as any form of cancer, hepatic insufficiency, or end-stage kidney disease), cognitive impairments, indications for revascularization or heart transplantation, or were under 18 years old. Each patient underwent a comprehensive clinical assessment, including a 6 min walk test (6MWT), electrocardiography (ECG), transthoracic echocardiography (TE), Holter ECG monitoring, coronary angiography, and blood tests. All participants received standard treatment according to current HF management guidelines [9].

Heart failure was classified as ischemic if significant coronary artery disease was present, defined by at least 50% stenosis in one or more of the major coronary arteries, as determined by coronary angiography or computed tomography angiography findings. Furthermore, myocardial infarction in anamnesis or previous revascularization procedures in the patient also signified ischemic heart failure. The cause of HF (whether ischemic, non-ischemic, or a combination of both) was further validated through cardiac magnetic resonance imaging and/or endomyocardial biopsy results.

### 2.2. Ethical Aspects

The research was conducted in alignment with the principles outlined in the Helsinki Declaration and adhered to Good Clinical Practice standards. The study protocol received approval from the Local Ethics Committee of the Cardiology Research Institute under protocol number 219, dated 26 October 2021. Prior to their inclusion in the study, all participants provided written informed consent. Consent for the publication of clinical data was obtained from all the subjects.

### 2.3. Clinical Assessment

For the analysis, we utilized two key measures—the walking distance in meters and the New York Heart Association (NYHA) functional class (FC) [10]. The walking distance was categorized based on the distance the patient was able to cover during the test, with specific thresholds corresponding to different levels of HF severity. Patients who were able to walk more than 551 m were considered to have no signs of HF. Those who covered a distance between 426 and 550 m were classified as having NYHA class I HF, indicating mild symptoms or none at all. A walking distance ranging from 301 to 425 m corresponded to NYHA class II, reflecting a slight limitation of physical activity. When the walking distance was between 151 and 300 m, patients were categorized as NYHA class III, indicating a marked limitation of activity. Finally, patients who walked less than 150 m were classified as NYHA class IV, which signifies severe limitations and symptoms even at rest.

Pre-implantation, standard 12-lead ECGs were obtained with the patient lying down, recorded at a paper speed of 25 mm/s and an amplitude of 10 mm/mV; these were subsequently analyzed. The gathered information encompassed the morphology of the bundle branches, the duration of the QRS complex, and the corrected QT interval, which was adjusted to consider any bundle branch block.

Echocardiographic evaluation, including the measurement of intracardiac hemodynamic parameters, was conducted utilizing a Philips HD15 PureWave ultrasound device (Philips Ultrasound, Inc., 22100 Bothell Everett Highway, Bothell, WA, 98021-8431, USA) prior to ICD implantation. The examination was performed from standard positions to evaluate the volume-dimensional indicators of the heart chambers and the left ventricular ejection fraction (LVEF). Additionally, the functionality of the mitral, tricuspid, and aortic valves was examined, as well as the contractility of both the right and left ventricles. The echocardiography was performed in accordance with current guidelines [11].

### 2.4. ICD Implantation and Programming

ICD implantation and programming was carried out in accordance with international standards [12]. The active-fixation defibrillation lead was placed at the septum or apex of the right ventricle (RV), while the atrial lead was positioned in the right atrial appendage. For patients eligible for cardiac resynchronization therapy, passive-fixation left ventricular leads were implanted in the lateral, posterolateral, or anterolateral cardiac veins. The lead positions were decided by the implanting physician in accordance with a standard protocol, using fluoroscopic guidance through a transvenous approach. The locations of the leads were confirmed using fluoroscopy from both postero-anterior and left anterior oblique views, along with intraoperative threshold testing.

### 2.5. Clinical Assessment, End Point, and Design of the Study

**Figure 1:**
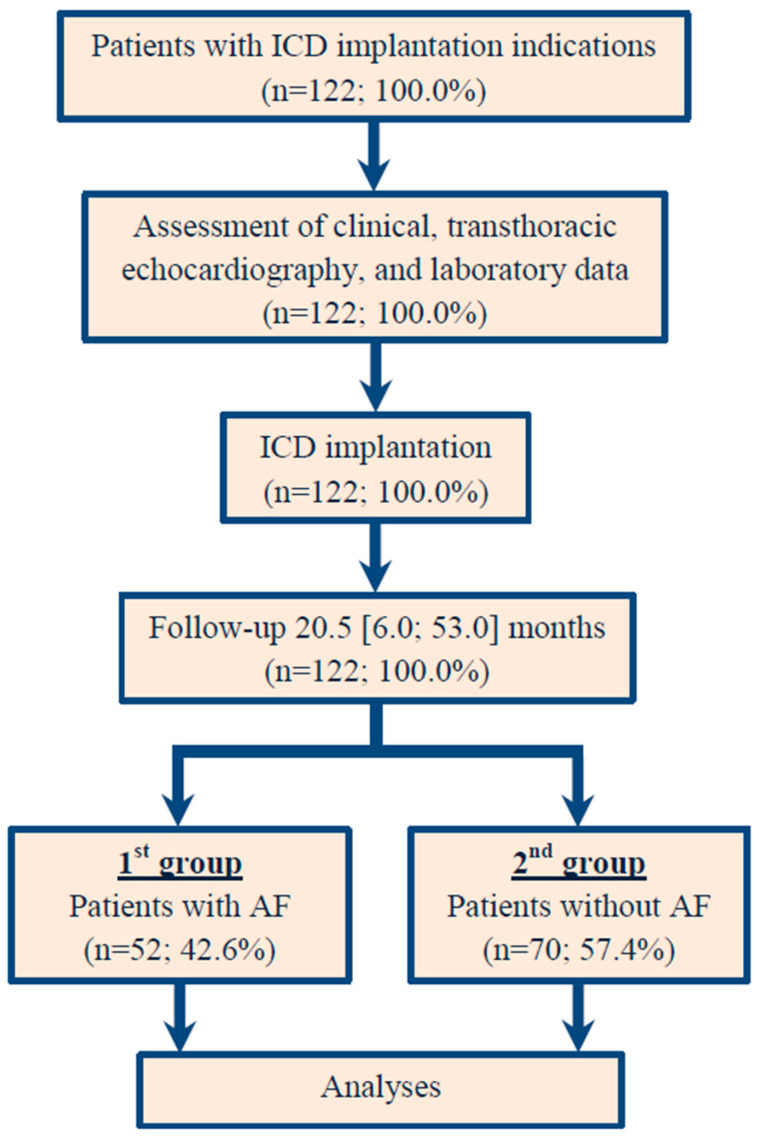
Study design and flow chart. AF: atrial fibrillation; ICD: implantable cardioverter–defibrillator.

Data on clinical evaluation were collected for all 122 patients involved in the study. The ECG, Holter ECG monitoring, or arrhythmic event reports recorded by the ICD were used to evaluate the primary endpoint. The primary endpoint was defined as AF. The participants were categorized into two groups based on the presence of AF either before the ICD implantation or during the follow-up period after the procedure. Clinical AF was defined as an ECG-documented heart rhythm with no discernible repeating P waves and irregular RR intervals with a duration of 30 or more seconds [13]. Subclinical AF or device-detected subclinical AF was defined as asymptomatic episodes of AF detected and confirmed by cardiac devices and monitors, and not previously detected by electrocardiographic or ambulatory monitoring [14]. Patients with a documented history of AF prior to device implantation were included, as well as those who developed AF during follow-up. The first group consisted of patients with AF, while the second group comprised those without AF. The study design and flow chart are illustrated in Figure 1.

### 2.6. Statistical Analysis, Risk Stratification, and Score Development

All statistical analyses were performed with software package Statistica (version 14.1.0., StatSoft Inc., Tulsa, OK, USA) and Medcalc (version 19.2.6, MedCalc Software, Ostend, Belgium). Statistical significance was defined by a *p*-value < 0.05. Categorical variables are presented as numbers (*n*) and percentages (%), and continuous variables are presented either as mean (M) ± standard deviation (SD) for normally distributed variables or as median (Me) and interquartile ranges [Q1; Q3] for non-normally distributed variables. The distribution of the continuous data of our entire ICD cohort was tested for normality using Kolmogorov–Smirnov and Shapiro–Wilk tests. The differences between groups for continuous data were evaluated using a two-sided Student’s *t*-test for normally distributed data or the Mann–Whitney U-test for ordinal or non-normally distributed data. For dependent samples, we applied the Wilcoxon test. The distribution of categorical variables was analyzed using chi-square or Fisher’s exact test. A comparison between patients with and without AF was performed for the complete dataset.

For the primary outcome, to identify the potential predictors of AF, we used a stepwise logistic regression. First, we applied a univariable logistic regression model to test the relationship between our primary endpoint (dependent variable) and all the clinical findings (independent variables). Characteristics significantly (*p* < 0.05) associated with the outcome in the univariable analysis were first entered as candidate variables in a multivariable logistic regression analysis. The included independent variables were tested for collinearity to exclude possible confounders. Significant correlations between the predictors and other measurements were examined using Spearman’s analysis. Goodness-of-fit was assessed by the Hosmer–Lemeshow test. Results from our regression analysis are presented as odds ratios (ORs) with 95% confidence intervals (CIs).

In the end, parameters independently associated with our endpoint were included in the risk stratification model. The area under the curve (AUC) was calculated to evaluate the discriminatory ability of the risk stratification score.

## 3. Results

### 3.1. Patient Baseline Demographic and Clinical Data

**Figure 2:**
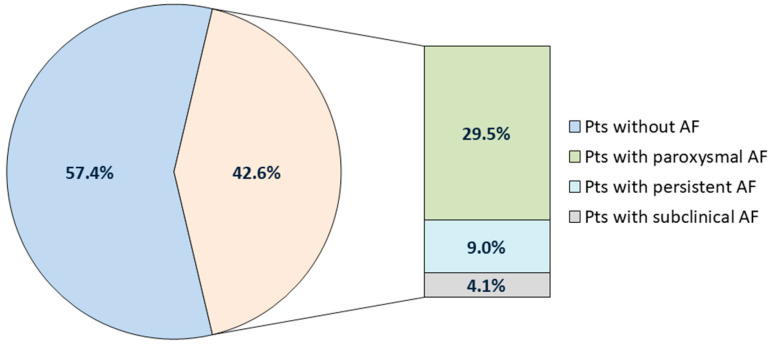
Incidence of different form of AF in our study cohort. Pts: patients; AF: atrial fibrillation.

In 122 (100.0%) patients with ICD implantation indications, the median age was 64.0 [58.0; 71.0] years; in total, 94 (77.0%) patients were males. The 1st group included 52 (42.6%) patients with clinical AF (paroxysmal AF [n = 36] and persistent AF [n = 11]) and subclinical AF registered for the first time according to ICD data (n = 5). Of these, seven patients (two patients with clinical AF and five with subclinical AF) had documented inappropriate ICD therapy. The prevalence of inappropriate ICD therapy related to AF in our cohort was 5.7% (7 patients out of 52). The 2nd group included 70 (57.4%) patients without AF. The incidence of different AF forms in our study cohort is presented in Figure 2.

The baseline characteristics of patients are presented in Table 1. There were no significant differences in baseline demographics and clinical features between patients with AF and those without, except for some of them. Patients with AF were significantly older than individuals without AF (69.0 [62.0; 75.5] years vs. 60.0 [56.0; 67.0] years; *p* < 0.001). There were no significant differences in systolic or diastolic blood pressure, body mass index, or estimated glomerular filtration rate between the two groups. Additionally, there were no significant differences in the history of myocardial infarction or coronary artery interventions. Furthermore, there were no significant variations regarding arrhythmias and bundle branch blocks prior to ICD implantation. Patients with AF had a higher likelihood of being diagnosed with sustained ventricular tachycardia and having indications for the secondary prevention of sudden cardiac death through ICD implantation (67.3% vs. 50.0%), although this difference did not reach statistical significance (*p* = 0.057). The groups did not show significant differences in cardiac medications, except for anticoagulants, antiplatelet agents, and angiotensin receptor neprilysin inhibitors. Patients with AF were more frequently prescribed anticoagulants (86.5% vs. 10.0%; *p* < 0.001) and less often prescribed antiplatelet agents (30.8% vs. 80.0%; *p* < 0.001), reflecting their history of AF and adherence to current management guidelines for the condition. Additionally, patients with AF were less likely to receive treatment with angiotensin receptor neprilysin inhibitors (ARNis) (4.1% vs. 15.6%; *p* = 0.016).

### 3.2. Patient Baseline Echocardiographic Characteristics

Based on the initial echocardiographic assessment (Table 2), ICD patients with AF were more likely to exhibit a larger left atrial index (LAI) (*p* = 0.002), which represents the size of the left atrium relative to body surface area, as well as a higher left ventricle early diastolic filling rate (LVE) (*p* = 0.006), increased right atrial index (RAI) (*p* = 0.019), and greater dimensions of both the left atrium and right ventricle (*p* = 0.031 and *p* = 0.016, respectively). They also showed increased thickness of the interventricular septum (IVS) (*p* = 0.016), larger volumes of the left atrium (LAV) and right atrium (RAV) (*p* = 0.011 and *p* = 0.028, respectively), elevated right ventricle systolic pressure (RVSP) (*p* = 0.017), and a higher ratio of LVE to left ventricle active filling rate (LVA) compared to patients without AF (*p* = 0.028). No significant differences were observed between the two groups in other pre-ICD echocardiographic parameters (Table 2).

### 3.3. Analysis for AF Risk Stratification

**Figure 3:**
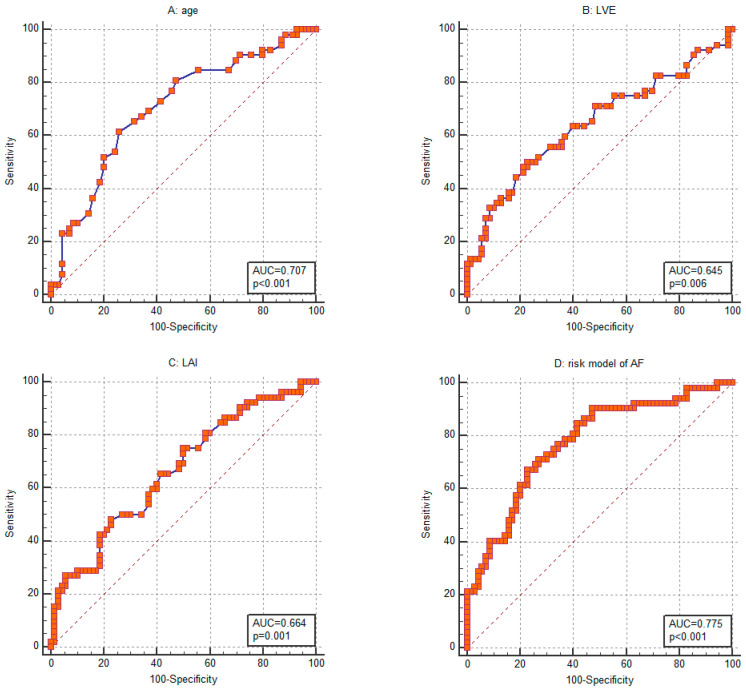
Receiver operating characteristic (ROC) curves for assessing the ability of (**A**) age, (**B**) left ventricle early diastolic filling rate (LVE), (**C**) left atrial index (LAI), and (**D**) the entire risk assessment model to distinguish between ICD patients with atrial fibrillation (AF) and those without.

Based on the analyzed parameters, age showed the strongest association with the incidence of AF. Its ability to distinguish AF cases was evaluated using ROC analysis, which yielded an AUC of 0.707 (95% CI: 0.617–0.786) (Figure 3A). For LVE, the ROC curve analysis demonstrated an AUC of 0.645 (95% CI: 0.553–0.729) (Figure 3B). Moreover, LVE was highly correlated with the LVE/LVA ratio (R = 0.750; *p* < 0.001). The LAI showed a comparable discriminative performance for AF occurrence, with an AUC of 0.664 (95% CI: 0.573–0.747) (Figure 3C). Additionally, LAI exhibited strong correlations with left atrial size (R = 0.915; *p* < 0.001), LAV (R = 0.940; *p* < 0.001), and RAV (R = 0.957; *p* < 0.001). Regarding the IVS, ROC analysis revealed an AUC of 0.627 (95% CI: 0.535–0.713). The discriminative abilities of RAI, RVSP, ARNi use, and NYHA class I were similar, with AUCs of 0.624 (95% CI: 0.531–0.710), 0.626 (95% CI: 0.533–0.712), 0.588 (95% CI: 0.495–0.676), and 0.578 (95% CI: 0.486–0.667), respectively.

**Figure 4:**
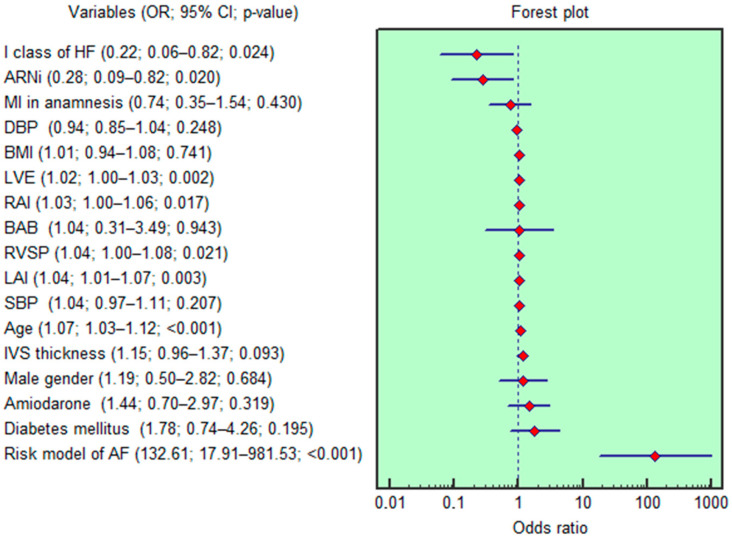
Forest plot illustrating the results of the univariable logistic regression analysis. Abbreviations—95% CI: 95% confidence interval; AF: atrial fibrillation; ARNi: angiotensin receptor neprilysin inhibitor; BABs: beta-adrenoblockers; BMI: body mass index; DBP: diastolic blood pressure; IVS: interventricular septum; HF: heart failure; LAI: left atrial index; LVE: left ventricle early diastolic filling rate; MI: myocardial infarction; OR: odds ratio; RAI: right atrial index; RVSP: right ventricle systolic pressure; SBP: systolic blood pressure.

The findings from the univariable logistic regression analysis are shown in Figure 4. Age (OR = 1.07; 95% CI 1.03–1.12; *p* < 0.001), NYHA class I (OR = 0.22; 95% CI 0.06–0.82; *p* = 0.011), RVSP (OR = 1.04; 95% CI 1.00–1.08; *p* = 0.016), LVE (OR = 1.02; 95% CI 1.00–1.03; *p* = 0.001), LAI (OR = 1.04; 95% CI 1.01–1.07; *p* < 0.001), RAI (OR = 1.03; 95% CI 1.00–1.06; *p* = 0.004), and the use of ARNi (OR = 0.28; 95% CI 0.09–0.82; *p* = 0.012) were each independently associated with the occurrence of AF in the univariable logistic regression analysis. However, after adjusting for factors such as gender, body mass index, history of myocardial infarction, diabetes mellitus, blood pressure, and treatment with beta-blockers and amiodarone, only three variables (age, LVE, and LAI) remained statistically significant in the multivariable regression analysis (OR = 1.08, 95% CI 1.03–1.12, *p* < 0.001; OR = 1.01, 95% CI 1.00–1.03, *p* = 0.026; OR = 1.03, 95% CI 1.00–1.06, *p* = 0.043, respectively).

### 3.4. Development of the AF Risk Score

A risk score for predicting the occurrence of AF was developed using logistic regression based on the gathered data. The final model included age, LVE, and LAI, as these factors continued to be significant in the multivariable logistic regression analysis, even after adjusting for gender and other established risk factors for AF. The AUC was calculated to assess the model’s ability to discriminate risk levels. The model showed a good discrimination capability with an AUC of 0.775 (Figure 3D). At a cutoff value of >0.47, the model achieved a sensitivity of 67.3% and a specificity of 77.2% in identifying HF patients with ICD at risk for AF. Our risk assessment demonstrated a negative predictive value of 80.0% and a positive predictive value of 59.6%. Overall, the risk model correctly classified 71.3% of cases.

The values for age (in years), LVE (in cm/s), and LAI (in mL/m^2^) need to be included in the scoring calculation. The result of the logistic equation below gives the probability (*p*) of experiencing atrial fibrillation. If the score exceeds 0.47, this risk score allows for the identification of HF patients with ICDs who are at a higher risk for AF. Accordingly, these patients should be subjected to intensified rhythm monitoring and improved device programming strategies to potentially reduce the frequency of inappropriate ICD therapy.

Equation (1): probability (*p*) of AF occurrence in HF patients with an ICD.

(1) $$p=\frac{1}{1+e^{-z}}$$

(2) $$z=-8.41+0.07\times \mathrm{age}\left[\mathrm{years}\right]+0.02\times \mathrm{LVE}[\mathrm{cm}/s]+0.03\times \mathrm{LAI}[\mathrm{mL}/m^{2}]\;$$

## 4. Discussion

The main findings of our study revealed that older age, a higher left ventricular early diastolic filling rate, and an increased left atrial index were independently associated with the occurrence of AF. By elucidating these relationships, we provided valuable insights that could inform clinical practice and enhance patient care.

Atrial fibrillation is a common arrhythmia that poses significant challenges in the management of HF patients, particularly those with ICDs. The findings underscore the complexity of AF’s interplay with HF and highlight critical predictors that can inform clinical practice. The prevalence of AF in our cohort (42.6%) aligns with the existing literature, which suggests that AF is notably common among HF patients, particularly those with reduced ejection fraction [1,15]. The age-related increase in AF incidence is well-documented and our results corroborate this trend, revealing that patients with AF are significantly older than their counterparts without AF. This finding is consistent with previous studies indicating that advancing age is a robust predictor of both AF and adverse outcomes in HF patients [5,6,16].

In addition to age, we have identified two echocardiographic parameters—left ventricular early diastolic filling rate and left atrial index—as significant predictors of AF. Atrial fibrillation and diastolic dysfunction are prevalent cardiac conditions that frequently coexist in HF patients. Their interrelationship is particularly significant in the context of patients who have ICDs. AF is characterized by chaotic electrical activity in the atria, leading to ineffective atrial contraction and an increased risk of thromboembolic events. In patients with HF, AF is common and can exacerbate symptoms, increase hospitalization rates, and worsen overall prognosis [1,15]. The presence of AF can lead to a rapid ventricular response, which may further compromise cardiac output, particularly in patients with underlying diastolic dysfunction. Diastolic dysfunction refers to the impaired ability of the ventricles to fill adequately during diastole. Diastolic dysfunction can result from various factors, including hypertension, ischemic heart disease, and aging. The impaired relaxation of the ventricles leads to increased left atrial pressure, which can contribute to the development of AF [17,18]. The relationship between AF and diastolic dysfunction is bidirectional. On the one hand, diastolic dysfunction can predispose patients to AF due to increased left atrial pressure and volume overload. Studies have shown that patients with elevated left atrial volumes are at a higher risk for developing AF [19,20]. On the other hand, once AF occurs, it can worsen diastolic function by eliminating effective atrial contraction, which plays a critical role in ventricular filling [21]. Our findings demonstrated that patients with AF exhibited significantly greater diastolic dysfunction based on LVE measurements compared to those without AF (74.5 [54.0; 97.5] cm/sec vs. 58.5 [48.0; 74.0] cm/s; *p* = 0.006). The univariable logistic regression showed that LVE (OR = 1.02; 95% CI 1.00–1.03; *p* = 0.001) was independently associated with AF. LVE remained significant in multivariable regression (OR = 1.01; 95% CI 1.00–1.03; *p* = 0.026), even after adjustment for gender and other well-known AF predictors. We used LVE in the risk stratification model because of its strong *p*-value. With the addition of this parameter in the risk score, the diastolic dysfunction processes should be properly represented.

The left atrial index is calculated by dividing the left atrial volume by body surface area. It provides a more accurate assessment of left atrial size relative to body size compared to absolute left atrial volume measurements. An elevated LAI is associated with increased left atrial pressure and reflects chronic pressure overload due to conditions such as hypertension or HF [19]. Studies have shown that an increased LAI is a strong predictor of adverse cardiovascular outcomes, including stroke and HF hospitalization [22]. In patients with AF, an enlarged left atrium may play a role in sustaining the arrhythmia as a result of structural remodeling and electrical alterations. An enlarged left atrium, indicated by an elevated LAI, predisposes patients to the development of AF. Increased left atrial pressure from diastolic dysfunction or volume overload can lead to the structural remodeling of the atrium, creating a substrate for AF [19]. In our study, patients with AF were more likely to have a larger LAI in comparison with individuals without AF (55.3 [49.3; 66.1] mL/m^2^ vs. 50.1 [40.6; 55.9] mL/m^2^; *p* = 0.002). According to the univariable and multivariable logistic regression, LAI was independently associated with AF, even after adjustment for gender and other well-known AF risk factors (OR = 1.03; 95% CI 1.00–1.06; *p* = 0.043). We used LAI in the risk stratification model because of its strong *p*-value. With the addition of this parameter in the risk score, the left atrium size should be properly represented.

Our multivariable logistic regression analysis has confirmed that these echocardiographic measures and age are independently associated with the occurrence of AF, reinforcing their importance in risk stratification for HF patients. The development of a logistic equation to predict AF based on these factors provides a valuable tool for clinicians. These factors are incorporated into the final predictive model, which demonstrates a strong discrimination capability with an AUC of 0.775. The model has achieved an accuracy of 71.3% in accurately diagnosing AF within our study population. Patients with an ICD who scored above the threshold value (0.47) are at an increased risk for AF. It is important to acknowledge that no score or model can guarantee a 100% probability of an individual experiencing AF. This predictive model can facilitate early intervention strategies aimed at preventing the onset of AF, thereby potentially reducing the incidence of inappropriate ICD therapies.

ICDs are commonly used in HF patients to prevent sudden cardiac death due to life-threatening arrhythmias. However, inappropriate shocks from ICDs can occur due to the misinterpretation of arrhythmias during episodes of AF. Rapid ventricular rates during AF may be mistaken for ventricular tachycardia or fibrillation, leading to unnecessary shocks that can negatively impact the patient’s quality of life [4,23]. The presence of an elevated LAI indicator may further complicate this issue. Patients with larger left atrial volumes often exhibit altered hemodynamics that could influence arrhythmogenesis. Therefore, understanding the interplay between these conditions is essential for optimizing ICD therapy. Several predictors have been identified for inappropriate ICD therapy in HF patients. These include older age, history of AF, renal impairment, and specific echocardiographic findings that are indicative of elevated LAI [24,25]. Identifying these predictors allows clinicians to better tailor ICD programming settings based on individual patient characteristics, which may help to reduce inappropriate shocks. Our study results showed that the prevalence of AF-related inappropriate ICD therapy in our cohort (5.7%) does not comply with results from the existing literature (25.4%) [26]. The low percentage of inappropriate ICD therapy in our study is most likely related to the fact that our study included patients with both primary and secondary prevention of sudden cardiac death. In contrast, a study by Lebedeva V. et al. included only patients with the primary prevention of SCD [26]. In our study, there were seven episodes of inappropriate shocks. This figure did not allow for a multivariable analysis, since at least ten events are required to estimate each variable included in the regression.

In conclusion, our study contributes to the growing body of evidence linking clinical and echocardiographic predictors to the incidence of AF in heart failure patients. By identifying older age, higher left ventricular early diastolic filling rate, and greater left atrial index as significant predictors, we provide insights that can enhance patient management strategies aimed at improving outcomes in this high-risk population. Our study offers a clinically useful prediction model for AF in HF patients with ICDs. Beyond device reprogramming, the findings could guide personalized monitoring, early pharmacological intervention, and advanced arrhythmia management to improve outcomes. Future studies should explore how integrating this model into remote monitoring systems and multidisciplinary HF care can optimize patient management.

### Study Limitations

We acknowledge that our research is a single-center study with a relatively small sample size; therefore, the results should be viewed with caution. This limitation is important, as the findings may not be generalizable to larger populations or different clinical settings. The small sample size diminishes the statistical power of our analyses, which could affect the reliability of the results. Additionally, since the study was conducted at a single center, the outcomes may be influenced by specific local practices, patient demographics, and other contextual factors that may not be present in other environments. As a result, while our findings provide preliminary insights, they should not be considered definitive. The proposed AF risk score shows an encouraging ability to distinguish outcomes within the current cohort; its generalizability to other populations has not been established, given the retrospective, single-center nature of the study. Our findings are preliminary and hypothesis-generating.

## 5. Conclusions

This study revealed that 42.6% of HF patients with ICDs experienced episodes of AF. Older age, higher left ventricular early diastolic filling rate, and greater left atrial index were identified as independent predictors of AF. The developed predictive model demonstrated a good discriminative ability for AF, effectively classifying 71.3% of cases.

## Figures and Tables

**Table 1:** Patient baseline clinical and demographic characteristics in the overall sample and in groups.

Demographic and Clinical Characteristics	Overall Population (n = 122)	1st Group Pts with AF (n = 52)	2nd Group Pts Without AF (n = 70)	*p* _2–3_
	1	2	3	
Age, year, Me [Q1; Q3]	64.0 [58.0; 71.0]	69.0 [62.0; 75.5]	60.0 [56.0; 67.0]	<0.001
Male gender, n (%)	94 (77.0)	41 (78.8)	53 (75.7)	0.687
Myocardial infarction in anamnesis, n (%)	73 (59.8)	29 (55.7)	44 (62.8)	0.433
Coronary artery stenting in anamnesis, n (%)	48 (39.3)	25 (48.1)	23 (32.8)	0.091
Systolic blood pressure, mmHg, M ± SD	120.7 ± 5.7	121.5 ± 4.6	120.2 ± 6.4	0.255
Diastolic blood pressure, mmHg, M ± SD	75.0 ± 3.7	74.5 ± 3.7	75.3 ± 3.6	0.237
Baseline body mass index, kg/m^2^, M ± SD	28.9 ± 5.1	29.1 ± 5.1	28.7 ± 5.2	0.954
Baseline eGFR, mL/min/1.73 m^2^, M ± SD	70.9 ± 19.2	67.1 ± 17.9	73.8 ± 19.8	0.109
Baseline New York Heart Association class:				
I, n (%)	18 (14.7)	3 (5.7)	15 (21.4)	0.016
II, n (%)	66 (54.1)	30 (57.7)	36 (51.4)	0.496
III, n (%)	38 (31.1)	19 (36.5)	19 (27.1)	0.271
Baseline 6 min walk test, m, M ± SD	353.9 ± 85.3	336.9 ± 78.1	366.5 ± 88.8	0.079
Bundle branch blocks and arrhythmias registered prior to ICD implantation:				
Left bundle branch block, n (%)	37 (30.3)	11 (21.1)	26 (37.1)	0.058
Right bundle branch block, n (%)	4 (3.3)	1 (1.9)	3 (4.3)	0.475
Sustained ventricular tachycardia, n (%)	70 (56.6)	35 (67.3)	35 (50.0)	0.057
Ventricular fibrillation, n (%)	6 (4.9)	1 (1.9)	5 (7.1)	0.191
ICD implantation indications:				
Primary prevention of SCD, n (%)	52 (42.6)	17 (32.7)	35 (50.0)	0.057
Secondary prevention of SCD, n (%)	70 (57.4)	35 (67.3)	35 (50.0)	0.057
Comorbidities:				
Diabetes mellitus, n (%)	26 (21.3)	14 (26.9)	12 (17.1)	0.195
Dyslipidemia, n (%)	61 (50.0)	27 (51.9)	34 (48.6)	0.717
Chronic obstructive pulmonary disease, n (%)	16 (13.1)	8 (15.4)	8 (11.4)	0.526
Stroke, n (%)	3 (2.4)	1 (1.9)	2 (2.8)	0.750
Baseline electrocardiographic findings:				
Corrected QT interval, ms, M ± SD	425.3 ± 33.0	431.7 ± 34.5	420.5 ± 31.2	0.109
QRS duration, ms, M ± SD	123.7 ± 36.1	116.5 ± 28.8	129.1 ± 40.0	0.156
Baseline therapy:				
Beta-adrenoblockers, n (%)	106 (86.9)	44 (84.6)	62 (88.6)	0.947
Loop diuretics, n (%)	50 (41.0)	24 (46.1)	26 (37.1)	0.320
Mineralocorticoid receptor antagonists, n (%)	79 (64.7)	36 (69.2)	43 (61.4)	0.376
Angiotensin-converting enzyme inhibitors, n (%)	72 (59.0)	34 (65.4)	38 (54.3)	0.092
Antiplatelet agents, n (%)	72 (59.0)	16 (30.8)	56 (80.0)	<0.001
Lipid-lowering treatment, n (%)	107 (87.7)	46 (88.5)	61 (87.1)	0.831
Angiotensin II receptor blocker, n (%)	24 (19.7)	12 (23.1)	12 (17.1)	0.419
Angiotensin receptor neprilysin inhibitors, n (%)	24 (19.7)	5 (4.1)	19 (15.6)	0.016
Amiodarone, n (%)	64 (52.4)	30 (57.7)	34 (48.6)	0.322
Sotalol, n (%)	4 (3.3)	3 (5.7)	1 (1.4)	0.187
Anticoagulants, n (%)	52 (42.6)	45 (86.5)	7 (10.0)	<0.001
Hypoglycemic drugs, n (%)	16 (13.1)	9 (17.3)	7 (10.0)	0.241
Sodium glucose co-transporter 2 inhibitors, n (%)	30 (24.6)	9 (17.3)	21 (30.0)	0.109

Values are expressed as M ± SD and Me [Q1; Q3] for continuous variables and n (%) for categorical variables. Abbreviations—AF: atrial fibrillation; ICD: implantable cardioverter–defibrillator; eGFR: estimated glomerular filtration rate; Pts: patients; SCD: sudden cardiac death.

**Table 2:** Pre-ICD implantation echocardiographic indicators.

Indicators	Overall Population (n = 122)	1st Group Pts with AF (n = 52)	2nd Group Pts Without AF (n = 70)	*p* _2–3_
	1	2	3	
RAI, mL/m^2^	39.4 [34.1; 45.7]	40.9 [35.8; 50.2]	37.9 [31.2; 43.1]	0.019
LAI, mL/m^2^	52.4 [45.2; 59.7]	55.3 [49.3; 66.1]	50.1 [40.6; 55.9]	0.002
LVEDI, mL/m^2^	85.9 [66.1; 118.6]	83.7 [65.8; 111.1]	86.7 [66.3; 124.9]	0.581
LVESI, mL/m^2^	51.5 [30.1; 77.5]	49.0 [30.2; 69.9]	55.0 [30.1; 87.8]	0.499
LVEF, %	40.0 [32.0; 55.0]	42.0 [32.5; 56.0]	40.0 [31.0; 53.0]	0.711
LVEDD, mm	60.5 [52.0; 67.0]	60.5 [51.5; 67.5]	60.5 [53.0; 66.0]	0.831
LVESD, mm	48.7 [38.0; 57.0]	48.2 [38.0; 56.5]	49.5 [38.0; 57.0]	0.781
LVEDV, mL	175.5 [128.0; 223.0]	171.0 [127.0; 219.0]	178.0 [130.0; 225.0]	0.541
LVESV, mL	105.0 [59.0; 150.0]	104.5 [59.5; 143.5]	111.0 [58.0; 154.0]	0.510
SV, mL	70.0 [59.0; 80.0]	69.0 [59.0; 79.5]	70.5 [59.0; 80.0]	0.652
MMI, g/m^2^	115.5 [99.0; 138.0]	116.0 [104.0; 142.0]	115.0 [97.0; 137.0]	0.294
CIB, L/min/m^2^	2.2 [1.8; 2.5]	2.1 [1.8; 2.6]	2.2 [1.9; 2.5]	0.688
LA, mm	45.0 [42.0; 50.0]	46.5 [43.0; 51.5]	45.0 [41.0; 48.0]	0.031
RV, mm	25.0 [22.0; 27.0]	25.5 [23.0; 28.0]	24.0 [22.0; 26.0]	0.016
RAV, mL	82.7 [67.3; 94.2]	85.5 [72.5; 102.3]	79.7 [65.4; 90.4]	0.028
LAV, mL	108.6 [92.0; 123.1]	113.7 [99.4; 129.9]	104.6 [86.1; 117.5]	0.011
LVSI	0.60 [0.55; 0.67]	0.61 [0.56; 0.66]	0.60 [0.54; 0.68]	0.995
LVE, cm/s	65.5 [50.0; 85.0]	74.5 [54.0; 97.5]	58.5 [48.0; 74.0]	0.006
LVA, cm/s	68.5 [57.0; 81.0]	68.0 [55.0; 82.0]	69.5 [60.0; 80.0]	0.663
LVE/LVA	0.90 [0.67; 1.42]	1.19 [0.69; 1.75]	0.79 [0.65; 1.18]	0.028
IVS, mm	10.5 [9.0; 12.0]	11.0 [10.0; 12.0]	10.0 [9.0; 11.5]	0.016
LVPW, mm	10.0 [9.0; 11.0]	10.5 [9.5; 11.1]	10.0 [9.0; 11.0]	0.295
RAA, cm/m^2^	1.8 [1.7; 1.9]	1.8 [1.7; 1.9]	1.7 [1.6; 1.9]	0.051
RVS, cm/m^2^	1.8 [1.7; 1.9]	1.8 [1.7; 1.9]	1.7 [1.6; 1.9]	0.084
RVSP, mmHg	30.0 [25.0; 36.0]	31.5 [27.0; 42.0]	30.0 [25.0; 33.0]	0.017

Values are expressed as Me [Q1; Q3] for continuous variables. Abbreviations—CIB: cardiac index in B mode; IVS: interventricular septum; LA: left atrium; LAI: left atrial index; LAV: left atrial volume; LVA: left ventricle active filling rate; LVE: left ventricle early diastolic filling rate; LVE/LVA: left ventricle early diastolic filling rate to left ventricle active filling rate ratio; LVEDD: left ventricular end-diastolic dimension; LVEDI: left ventricular end-diastolic index; LVEDV: left ventricular end-diastolic volume; LVEF: left ventricle ejection fraction; LVESD: left ventricular end-systolic dimension; LVESI: left ventricular end-systolic index; LVESV: left ventricular end-systolic volume; LVPW: left ventricle posterior wall; LVSI: left ventricular sphericity index; MMI: myocardial mass index; RAA: ratio of ascending aortic diameter to body surface area; RAI: right atrial index; RAV: right atrial volume; RV: right ventricle; RVS: ratio of the Valsalva sinus diameter to the body surface area; RVSP: right ventricle systolic pressure; SV: stroke volume.

## Data Availability

Available upon request.

## Abbreviations

The following abbreviations are used in this manuscript:

6MWT	Six Minute Walk Test
AF	Atrial Fibrillation
ARNi	Angiotensin Receptor Neprilysin inhibitor
AUC	Area Under the ROC Curve
CI	Confidence Interval
ECG	Electrocardiography
FC	Functional Class
HF	Heart Failure
ICD	Implantable Cardiac–Defibrillator
LAI	Left Atrial Index
LAV	Left Atrial Volume
LVA	Left Ventricle Active Filling Rate
LVE	Left Ventricle Early Diastolic Filling Rate
LVEF	Left Ventricle Ejection Fraction
M	Mean
Me	Median
MI	Myocardial Infarction
NYHA	New York Heart Association
OR	Odds ratio
RAI	Right Atrial Index
RAV	Right Atrial Volume
RV	Right Ventricle
RVSP	Right Ventricle Systolic Pressure
SD	Standard Deviation
TE	Transthoracic Echocardiography
VT	Ventricular Tachycardia
